# Left heart function and strain for predicting change in hemoglobin levels in pediatric kidney transplantation recipients

**DOI:** 10.3389/fped.2025.1452928

**Published:** 2025-03-21

**Authors:** Shufan Yue, Fei Xiao, Rui Fan, Wei Li, Donghong Liu, Fengjuan Yao, Hong Lin, Chenglin Wu, Longshan Liu, Changxi Wang, Jun Li, Cuiling Li

**Affiliations:** ^1^ Department of Medical Ultrasonics, Institute of Diagnostic and Interventional Ultrasound, The First Affiliated Hospital of Sun Yat-Sen University, Guangzhou, China; ^2^ Department of Organ Transplantation, The First Affiliated Hospital of Sun Yat-Sen University, Guangzhou, China; ^3^ Department of Medical Ultrasonics, Guangxi Hospital Division of the First Affiliated Hospital, Sun Yat-Sen University, Nanning, China

**Keywords:** cardiac function, kidney transplantation, anemia, hemoglobin, pediatric

## Abstract

**Introduction:**

Anemia is prevalent after kidney transplantation (KTx) and is associated with reduced graft survival. The associations between temporal changes in hemoglobin (Hb) level in the early posttransplant period with left ventricular (LV) and atrial (LA) function and strain are unknown.

**Methods:**

The study cohort included 71 successful pediatric KTx recipients between January 2021 and September 2022. Echocardiography was used to evaluate the cardiac structure, function, and strain both before and after KTx. Temporal changes in Hb values within 6 months after KTx were evaluated. According to the LV mass index (LVMI), recipients were divided into a left ventricular hypertrophy (LVH) group and a non-LVH group.

**Results:**

Before KTx, the LVH group had a lower level of Hb and a higher incidence of anemia than the non-LVH group. However, this difference between the groups disappeared after KTx. Changes in Hb were faster in the LVH group than in the non-LVH group. There was a negative association between the absence of an Hb increase and diastolic blood pressure, the LVMI, early diastolic mitral annulus velocity to early diastolic filling wave ratio (E/E’), and the left atrial emptying volume index (LAEVI), while there was a positive association between LV ejection fraction (LVEF), LV global longitudinal strain, and LA strain. The LVMI and changes in LVEF showed a negative correlation, whereas changes in the LAEVI showed a positive correlation with the absence of Hb increase during the early period after KTx.

**Conclusion:**

Children with LVH have a lower level of Hb before KTx and a higher level of Hb increase in the early postoperative period following KTx. LVMI and changes in LVEF and LAEVI have predictive value in absence of Hb increase in pediatric KTx recipients.

## 1 Introduction

Anemia after kidney transplantation (PTA) is common following kidney transplantation (KTx). The prevalence rate of PTA ranges from 36% to 52% (1–3). In early PTA, anemia occurs within 6 months of transplantation, while in late PTA, anemia occurs more than 6 months following transplantation (4). It is notable that the severity of PTA correlates with the degree to which the KTx recipient's renal function is compromised (5). In addition, PTA has been associated with an increased mortality rate and decreased graft function (2, 4). Furthermore, cardiovascular disease is a frequent complication after kidney transplantation and represents one of the leading causes of mortality (6). In addition, during the early postoperative period following transplantation, there was an association between the absence of hemoglobin (Hb) increase and the risk of graft loss (7).

Changes in Hb levels and the severity of PTA are closely related to changes in cardiac structure and function (8). In addition, increasing the preload and reducing the afterload in patients with chronic PTA could result in hyperdynamic states and an increase in the cardiac output; however, this could lead to left ventricular hypertrophy (LVH) and maladaptive LVH. Therefore, PTA is a risk factor for cardiovascular disease events and all-cause death (9). PTA has also been found to be an independent risk factor for cardiovascular diseases, such as heart failure (10). Previous studies have found changes in cardiac structure and function after transplantation, especially in the left ventricle (LV) (11–14). Therefore, it is pertinent to examine PTA and the corresponding changes in Hb levels.

Global longitudinal peak systolic left ventricular strain (LV-GLS) is a significant independent predictor of heart failure hospitalization or cardiovascular mortality after KTx (15). After KTx, pediatric patients often develop LVH (16). Adult renal transplant recipients with persistent LVH may experience higher incidences of infections and chronic rejection, which worsens their prognosis (17). However, studies on the left atrium (LA) in KTx are still limited, and further research is needed. Regele et al. found that in patients who underwent kidney transplantation, the risk of mortality was lower on both a general and cardiovascular basis (14). It was reported that in patients with chronic kidney disease (CKD), LA strain (LASr) was associated with adverse cardiovascular outcomes; hence, PTA is a promising factor for predicting cardiovascular events and assessing risk (18). Nevertheless, there is currently no relevant report on posttransplant LASr and the outcomes of kidney transplantation. However, as most of those studies focused primarily on adult patients, data on pediatric patients are limited.

Currently, the relationship between left heart strain and function and Hb levels and Hb increase after KTx is still unclear. The aim of the study was to examine the relationship between temporal changes in Hb during the early posttransplant period and LV and LA function and strain in pediatric KTx recipients.

## 2 Materials and methods

### 2.1 Study population

Between January 2021 and September 2022, 71 children with end-stage kidney disease (ESKD) who received kidney transplants were recruited from the First Affiliated Hospital of Sun Yat-Sen University (SYSU). All patients in this study underwent deceased donor kidney transplantation. The study was conducted in accordance with the principles of the Declaration of Helsinki. The study was approved by SYSU's ethics committee, and written informed consent was obtained from all the patients enrolled in the study. All parents gave informed consent. The follow-up duration with echocardiography after transplantation ranged from 3 to 5 months, with an average follow-up time of 4.17 months.

The study's inclusion criteria were as follows: (1) patients must be less than 18 years of age; (2) have been approved for KTx; (3) have normal sinus rhythm; (4) not have congenital cardiovascular diseases; and (5) have had echocardiographic evaluations before KTx. The exclusion criteria were as follows: (1) patients with congenital heart disease; (2) patients lost to follow-up after KTx; (3) poor echocardiography images; and (4) a lack of recognizable R-R interval on echocardiography. The diagnosis of hypertension in children was based on the criteria defined by the Chinese guidelines for the management of hypertension (2018 revised edition) (19).

In total, 37 healthy children, who were well-matched by age, gender, and body mass index (BMI) with the patients at their post-KTx visit and who did not have any history of cardiovascular or renal disease, were enrolled as the control group. In addition, the participants in the control group did not have cardiovascular diseases, cardiovascular risk factors (including hypertension), hemodynamically significant cardiovascular abnormalities, active diseases that could interfere with cardiac function, or metabolic disease.

### 2.2 Clinical parameters

Demographic parameters (age, gender, height, and weight) and clinical variables, including heart rate, blood pressure (BP), causes of CKD, and duration of dialysis before KTx, were collected. The Mosteller formula was used to calculate body surface area (BSA). The BMI was calculated by dividing weight (kg) by the square of height (m^2^). Hb, urea, and creatinine levels were assessed on the day of or the day before surgery, and at 6 months postoperatively, with an average time of 6.03 months. In this study, the estimated glomerular filtration rate (eGFR) was calculated using the modified Schwartz's formula: eGFR (mL/min/1.73 m^2^) = [0.413 × height (cm)]/serum creatinine (mg/dL).

Anemia was defined according to gender and age, based on the KDIGO (Kidney Disease: Improving Global Outcomes) guidelines for patients with CKD (20). A diagnosis of anemia was made if the Hb concentration was <110 g/L in children aged 0.5–5 years, <115 g/L in children aged 5–12 years, and <120 g/L in children aged 12–15 years. Children aged >15 years were diagnosed with anemia if their Hb concentration was below 130 g/L for boys or below 120 g/L for girls. The absence of Hb increase was defined as less than 5 g/L/month after kidney transplantation, in accordance with previous studies (7).

### 2.3 Echocardiography

Separate echocardiographic recordings were obtained for the pre-KTx period (on the day of or the day before KTx) and the post-KTx period (3–5 months after KTx). Echocardiography with two-dimensional imaging, tissue doppler imaging, and speckle tracking echocardiography were performed on all participants in accordance with current guidelines and recommendations. Standard apical four-, two-, and three-chamber views were recorded for analysis of the LV, and apical four-, and two-chamber views were used for the LA analysis. The LV mass index (LVMI) was calculated by dividing LV mass with the patient's BSA [LVMI = LV mass (g)/BSA (m^2^)]. The presence of LVH was determined using the 95th percentile of the LVMI (21). The recipients were further divided into groups according to their LVMI before KTx based on the presence of LVH, since LVH was considered a surrogate marker for target organ damage. Using the Simpson method, the LV ejection fraction (EF) was measured from the apical four- and two-chamber views. Using two-dimensional echocardiography in the parasternal long-axis view, the LVEF, end-diastolic volume (EDV), and end-systolic volume (ESV) were measured. The Mosteller formula was used to index the LA and LV volumes and the LV mass to the body surface area.

After using the biplane area-length method to assess the volume of the left atrium, the left atrial volume index (LAVI) was calculated by dividing the volume of the left atrium by the BSA. The following LA volumes were determined and indexed to the BSA: maximum LA volume index (LAVImax), minimum LA volume (LAVImin), volume index before atrial contraction (LAVIpreA), and the atrial volume in the last frame before or at the time of the P wave. The diastolic emptying index (DEI) was calculated using the following formula:

[(LAVImax−LAVImin)/LAVImax]×100%.

The passive emptying index (PEI) was determined using the formula:

[(LAVImax−LAVIpreA)/LAVImax]×100%;

The active emptying index (AEI) was determined using the formula:

[(LAVIpreA−LAVImin)/LAVIpreA]×100%

The LA ejection fraction (LAEF) was calculated using the equation:

LAEF=(LAVImax−LAVImin)/LAVImax×100%.

The LA emptying volume index (LAEVI) was calculated using the equation:

LAEVI=LAVImax−LAVImin.

The early (E) and late (A) diastolic peak velocities of the mitral inflow were measured using pulsed wave Doppler, and their ratio (E/A) was calculated. Tissue Doppler imaging was also used to assess the early diastolic peak velocity of both the septal and lateral mitral valve annuli. Mitral valve annular peak diastolic velocity was calculated as the average of the septal and lateral annular values (E’ average). The E/E’ average was also calculated.

### 2.4 Statistical analysis

SPSS version 25.0 (IBM Corp., Armonk, NY, USA) was used for statistical analysis. Frequencies (percentages) were used to express categorical variables, while continuous variables were reported as means and standard deviations. Categorical variables were compared using the chi-square test or Fisher's exact test. For continuous variables, Student’s *t*-test or paired Student’s *t*-test and the Kruskal–Wallis test were used. Preoperative and postoperative variables were compared by paired *t*-test. The linear relationship between the variables was assessed using Pearson’s or Spearman’s correlation coefficients. Both univariate and multivariate linear regression analyses were performed to determine the relationship between changes in clinical factors and ultrasonographic parameters. Using the multivariable stepwise logistic regression model, changes in clinical factors were also linked to changes in ultrasonic parameters. For the multivariate analysis, factors with *p* < 0.05 were selected as associated variables. Furthermore, two-sided *p*-values < 0.05 were deemed statistically significant.

## 3 Results

### 3.1 Baseline characteristics

A total of 71 children who underwent kidney transplantation and 37 appropriately matched healthy controls were included in this study. The probability of congenital anomalies of the kidney and urinary tract (CAKUT) as the cause of ESKD was higher in the LVH group compared to the non-LVH group (Supplementary Table S1). Most of the included patients were on triple immunosuppressants, including calcineurin inhibitors, prednisone, and mycophenolate mofetil or mycophenolic acid. Only three patients were treated with a combination of two immunosuppressive agents. As shown in Table 1, the patients’ age, height, weight, and BSA increased after KTx. Systolic and diastolic BP (SBP and DBP) levels at the post-KTx visit decreased postoperatively but were still higher than those of the control group (*p* < 0.001). There was no significant difference in age, gender, height, weight, BMI, and BSA between patients at the post-KTx visit and the controls. Among the transplant recipients, 13 (18.3%) developed infections after transplantation, and four (5.6%) experienced rejection within 6 months after surgery (Supplementary Table S1). The Hb level increased from 97.97 ± 20.37 to 118.21 ± 14.19 g/L after KTx (*p* < 0.001). The incidence of anemia decreased from 87.1% (*n* = 58) to 50.7% (*n* = 36) among the children who received a KTx (*p* < 0.001). In addition, the LVMI and the prevalence of LVH were significantly decreased after KTx (*p* < 0.01). No significant differences were found in E/A, LV-GLS, LASct, LAEF, and DEI between the pre- and post-KTx values. In addition, after KTx, some cardiac echocardiographic parameters were still worse at the post-KTx visit in the patients than in the control group.

**Table 1:** The comparison of the clinical and echocardiographic characteristics of the patients’ pre- and post-KTx visits and the control group.

Variables	Pre-KTx visit	Post-KTx visit	*p*-Value^a^	Control group	*p-*Value^b^
Clinical characteristics					
Age (years)	10.14 ± 4.04	10.44 ± 4.09	**<0**.**001**	9.90 ± 3.43	0.497
Male [*n* (%)]	44 (62.0)		**—**	21 (56.8)	0.599
Height (cm)	131.77 ± 24.06	133.32 ± 23.52	**0**.**001**	136.52 ± 21.68	0.478
Weight (kg)	29.44 ± 13.81	31.42 ± 14.56	**0**.**006**	34.61 ± 14.54	0.283
BMI (kg/m^2^)	15.95 ± 2.89	16.53 ± 3.95	0.169	17.70 ± 3.36	0.128
BSA (m^2^)	1.04 ± 0.33	1.07 ± 0.33	**0**.**002**	1.14 ± 0.33	0.325
Office SBP (mmHg)	124.86 ± 18.34	112.69 ± 11.67	**<0**.**001**	98.43 ± 12.85	**<0**.**001**
Office DBP (mmHg)	84.92 ± 15.19	74.58 ± 11.09	**<0**.**001**	60.65 ± 8.08	**<0**.**001**
Hypertension [*n* (%)]	53 (74.6)	29（40.8）	**<0**.**001**	—	—
Heart Rate (bpm)	87.03 ± 15.61	88.19 ± 15.73	0.975	80.05 ± 16.15	**0**.**016**
Hb (g/L)	97.97 ± 20.37	118.21 ± 14.19	**<0**.**001**	—	—
Anemia [*n* (%)]	58 (81.7)	36 (50.7)	**<0**.**001**	—	—
Urea (μmol/L)	26.19 ± 10.04	7.48 ± 3.02	**<0**.**001**	—	—
Creatinine (μmol/L)	818.17 ± 419.73	70.27 ± 24.86	**<0**.**001**	—	—
eGFR (mL/min/1.73 m^2^)	5.25 ± 2.58	60.09 ± 18.81	**<0**.**001**	—	—
Echocardiographic parameters					
E/A	1.42 ± 0.46	1.38 ± 0.37	0.735	1.90 ± 0.50	**<0**.**001**
E/E'	9.63 ± 2.39	8.66 ± 1.88	**0**.**004**	6.23 ± 1.01	**<0**.**001**
LVMI (g/m^2^)	106.84 ± 46.34	76.83 ± 24.41	**<0**.**001**	61.15 ± 13.92	**<0**.**001**
LVH [*n* (%)]	43（60.6）	24（33.8）	**0**.**002**	—	—
LV-EDVI (mL/m^2^)	61.13 ± 32.62	49.73 ± 18.40	**0**.**012**	48.50 ± 15.92	0.731
LV-ESVI (mL/m^2^)	29.28 ± 22.41	21.37 ± 10.69	**0**.**004**	16.99 ± 5.77	**0**.**022**
LVEF (%)	55.03 ± 10.21	57.90 ± 10.88	**0**.**025**	64.76 ± 5.31	**<0**.**001**
LV-GLS (%)	−16.64 ± 3.72	−17.55 ± 3.90	0.064	−21.14 ± 2.36	**<0**.**001**
LASr (%)	33.27 ± 8.45	36.01 ± 7.29	**0**.**008**	46.32 ± 7.55	**<0**.**001**
LAScd (%)	17.20 ± 9.07	21.27 ± 8.20	**<0**.**001**	34.03 ± 5.70	**<0**.**001**
LASct (%)	16.01 ± 7.84	14.79 ± 6.87	0.210	12.35 ± 3.37	**0**.**045**
LAEF (%)	65.36 ± 9.29	66.70 ± 7.44	0.244	74.07 ± 5.90	**<0**.**001**
LAEVI (mL/m^2^)	14.18 ± 7.12	10.96 ± 3.70	**<0**.**001**	10.62 ± 3.16	0.634
LAVImax (mL/m^2^)	22.44 ± 12.28	16.36 ± 4.84	**<0**.**001**	14.41 ± 4.09	**0**.**039**
LAVImin (mL/m^2^)	8.20 ± 6.31	5.47 ± 1.89	**<0**.**001**	3.81 ± 1.35	**<0**.**001**
LAVIpreA (mL/m^2^)	14.71 ± 9.92	9.49 ± 3.35	**<0**.**001**	6.23 ± 2.24	**<0**.**001**
DEI (%)	65.42 ± 9.79	66.29 ± 7.56	0.463	73.47 ± 6.09	**<0**.**001**
PEI (%)	35.94 ± .16.29	41.92 ± 13.16	**0**.**002**	57.16 ± 7.25	**<0**.**001**
AEI (%)	29.48 ± 14.65	24.36 ± 12.52	**0**.**008**	16.30 ± 4.53	**<0**.**001**

KTx, kidney transplantation; BMI, body mass index; SBP, systolic blood pressure; DBP, diastolic blood pressure; Hb, hemoglobin; eGFR, estimated filtration rate; LV, left ventricle; LA, left atrium; LVMI, LV mass index; LVH, left ventricular hypertrophy; EDVI, end-diastolic volume index; ESVI, end-systolic volume index; EF, ejection fraction; E and A, mitral peak E- and A-wave velocity; E′, pulsed-wave TDI-derived mitral annular early diastolic velocity; LAVI, left atrial volume index; LASr, LAScd, and LASct, left atrial reservoir, conduit, and contraction strain; LV-GLS, global longitudinal peak systolic left ventricular strain; DEI, diastolic emptying index; PEI, passive emptying index; AEI, active emptying index. Bold values indicate that *p*-values < 0.05.

^a^ Pre-KTx visit vs. post-KTx visit.

^b^ Control group vs. post-KTx visit.

### 3.2 Clinical and echocardiographic characteristics pre- and post-KTx according to LVMI categories

As shown in Table 2, gender distribution differed between the LVH group and the non-LVH group (*p* = 0.006). Compared with the non-LVH group, the LVH group had higher pre-KTx LVMI, LV-EDVI, and LV-ESVI, and worse LVEF. Pre-KTx SBP and DBP were lower in the non-LVH group than the LVH group (*p* < 0.001). The incidence of hypertension was also lower in the non-LVH group (57.1% vs. 86.0%, *p* = 0.011) than in the LVH group. Before KTx, the prevalence of anemia was higher in the LVH group than in the non-LVH group (67.9% vs. 90.7%, *p* < 0.01), with a lower level of Hb (106.75 ± 17.54 g/L vs. 92.26 ± 20.23 g/L, *p* < 0.05). No significant differences were observed in urea, creatinine, and eGFR. Furthermore, lower LVEF, LV-GLS, LASr, LAScd, and LAEF were detected in the LVH group than in the non-LVH group (*p* < 0.05). The LAEVI, LAVImax, LAVImin, and LAVIpreA were higher in the LVH group than in the non-LVH group (*p* < 0.001). DEI and PEI were lower in the LVH group than the non-LVH group (*p* < 0.05).

**Table 2:** The clinical and echocardiographic characteristics before and after KTx according to left ventricular mass index categories before KTx.

Variables	Pre-KTx visit			Post-KTx visit		
	Non-LVH group (*n* = 28)	LVH group (*n* = 43)	*p*-Value	Non-LVH group (*n* = 28)	LVH group (*n* = 43)	*p*-Value
Clinical characteristics						
Age (years)	10.05 ± 3.82	10.20 ± 4.22	0.882	—	—	—
Male [*n* (%)]	23 (82.1)	21 (48.8)	**0**.**006**	—	—	—
Duration of CKD (months)	32.85 ± 20.65	28.49 ± 33.59	0.541	—	—	—
Dialysis [*n* (%)]	23 (82.1)	37 (86.0)	0.742	—	—	—
Duration of dialysis (months)	13.99 ± 14.02	11.00 ± 12.93	0.360	—	—	—
Ratio of dialysis duration to CKD duration	0.54 ± 0.41	0.68 ± 0.34	0.143	—	—	—
Height (cm)	132.68 ± 24.27	131.19 ± 24.19	0.801	134.25 ± 24.32	132.72 ± 23.25	0.791
Weight (kg)	29.98 ± 14.12	29.09 ± 13.75	0.793	32.95 ± 14.87	30.43 ± 14.44	0.479
BMI (kg/m^2^)	15.85 ± 3.02	16.02 ± 2.83	0.806	17.38 ± 3.96	15.98 ± 3.89	0.144
BSA (m^2^)	1.06 ± 0.34	1.03 ± 0.33	0.761	1.10 ± 0.34	1.05 ± 0.33	0.589
Hypertension [*n* (%)]	16 (57.1)	37 (86.0)	**0**.**011**	—	—	—
Office SBP (mmHg)	113.18 ± 15.84	132.47 ± 15.78	**<0**.**001**	109.57 ± 9.53	114.72 ± 12.57	0.069
Office DBP (mmHg)	76.93 ± 13.03	90.12 ± 14.32	**<0**.**001**	71.04 ± 10.53	76.88 ± 10.95	**0**.**029**
HR (bpm)	89.23 ± 15.44	85.60 ± 15.77	0.380	89.11 ± 16.51	87.55 ± 15.37	0.706
Hb (g/L)	106.75 ± 17.54	92.26 ± 20.23	**0**.**003**	118.46 ± 10.37	118.05 ± 16.31	0.905
Anemia [*n* (%)]	19 (67.9)	39 (90.7)	**0**.**026**	12 (42.9)	24 (55.8)	0.286
Urea (μmol/L)	26.84 ± 9.78	25.76 ± 10.29	0.660	7.41 ± 2.22	7.53 ± 3.48	0.871
Creatinine (μmol/L)	771.46 ± 270.24	848.58 ± 494.24	0.453	75.86 ± 28.33	66.63 ± 21.91	0.127
eGFR (mL/min/1.73 m^2^)	5.59 ± 2.86	5.04 ± 2.41	0.387	58.29 ± 19.29	61.27 ± 18.63	0.519
Echocardiographic parameters						
E/A	1.52 ± 0.41	1.36 ± 0.48	0.167	1.31 ± 0.22	1.42 ± 0.44	0.270
E/E'	8.43 ± 1.60	10.42 ± 2.51	**<0**.**001**	8.23 ± 1.61	8.94 ± 2.01	0.122
LVMI (g/m^2^)	69.70 ± 12.83	131.02 ± 44.21	**<0**.**001**	66.07 ± 20.93	83.85 ± 24.16	**0**.**002**
LV-EDVI (mL/m^2^)	49.59 ± 34.25	68.64 ± 29.55	**0**.**015**	44.54 ± 12.03	53.11 ± 21.01	0.055
LV-ESVI (mL/m^2^)	20.97 ± 14.98	34.68 ± 24.83	**0**.**011**	19.47 ± 7.40	22.60 ± 12.31	0.230
LV EF (%)	58.43 ± 7.57	52.82 ± 11.15	**0**.**023**	56.76 ± 10.85	58.64 ± 10.96	0.479
LV-GLS (%)	−17.99 ± 3.45	−15.76 ± 3.66	**0**.**012**	−18.89 ± 3.07	−16.68 ± 4.16	**0**.**019**
LASr (%)	37.07 ± 8.24	30.79 ± 7.71	**0**.**002**	39.18 ± 7.82	33.95 ± 6.18	**0**.**003**
LAScd (%)	20.36 ± 9.58	15.14 ± 8.19	**0**.**017**	23.00 ± 9.90	20.14 ± 6.77	0.152
LASct (%)	16.75 ± 8.46	15.53 ± 7.47	0.527	16.32 ± 7.97	13.79 ± 5.93	0.130
LAEF (%)	69.16 ± 7.57	62.88 ± 9.54	**0**.**005**	70.80 ± 7.79	64.03 ± 5.89	**<0**.**001**
LAEVI (mL/m^2^)	9.97 ± 3.58	16.91 ± 7.53	**<0**.**001**	10.87 ± 4.38	11.03 ± 3.24	0.858
LAVImax (mL/m^2^)	14.42 ± 4.11	27.66 ± 13.03	**<0**.**001**	15.26 ± 5.39	17.07 ± 4.36	0.123
LAVImin (mL/m^2^)	4.32 ± 1.39	10.73 ± 6.96	**<0**.**001**	4.41 ± 1.62	6.17 ± 1.74	**<0**.**001**
LAVIpreA (mL/m^2^)	8.31 ± 2.84	18.88 ± 10.66	**<0**.**001**	8.59 ± 3.58	10.07 ± 3.09	0.067
DEI (%)	69.51 ± 8.26	62.76 ± 9.87	**0**.**004**	70.57 ± 7.61	63.50 ± 6.16	**<0**.**001**
PEI (%)	41.46 ± 17.49	32.35 ± 14.57	**0**.**020**	43.76 ± 15.63	40.73 ± 11.31	0.347
AEI (%)	28.06 ± 16.81	30.41 ± 13.19	0.512	26.81 ± 15.62	22.77 ± 9.88	0.185

KTx, kidney transplantation; BMI, body mass index; SBP, systolic blood pressure; DBP, diastolic blood pressure; Hb, hemoglobin; eGFR, estimated filtration rate; LV, left ventricle; LA, left atrium; LVMI, LV mass index; LVH, left ventricular hypertrophy; EDVI, end-diastolic volume index; ESVI, end-systolic volume index; EF, ejection fraction; E and A, mitral peak E- and A-wave velocity; E′, pulsed-wave TDI-derived mitral annular early diastolic velocity; LAVI, left atrial volume index; LASr, LAScd, and LASct, left atrial reservoir, conduit, and contraction strain; LV-GLS, global longitudinal peak systolic left ventricular strain; DEI, diastolic emptying index; PEI, passive emptying index; AEI, active emptying index; CKD, chronic kidney disease. Bold values indicate that *p*-values < 0.05.

After KTx, there was no significant difference in the prevalence of anemia and Hb levels between the LVH group and the non-LVH group (*p* = 0.286 and *p* = 0.905, Table 2). The LVH group still had a higher LVMI and higher prevalence of LVH than the non-LVH group after KTx (66.07 ± 20.93 g/m^2^ vs. 83.85 ± 24.16 g/m^2^, *p* = 0.002; 17.9% vs. 44.2%, *p* = 0.039). Better DBP, LV-GLS, LASr, and LAEF were found in the non-LVH group than the LVH group. After KTx, the LAEVI in the LVH group was comparable to that in the non-LVH group (*p* = 0.858), as was the LAVImax (*p* = 0.123) and LAVIpreA (*p* = 0.067), while the LAVImin was still higher in LVH group in comparison to that in the non-LVH group (*p* < 0.001). Furthermore, compared with the non-LVH group, a lower DEI was detected in the LVH group (*p* < 0.001).

**Figure 1:**
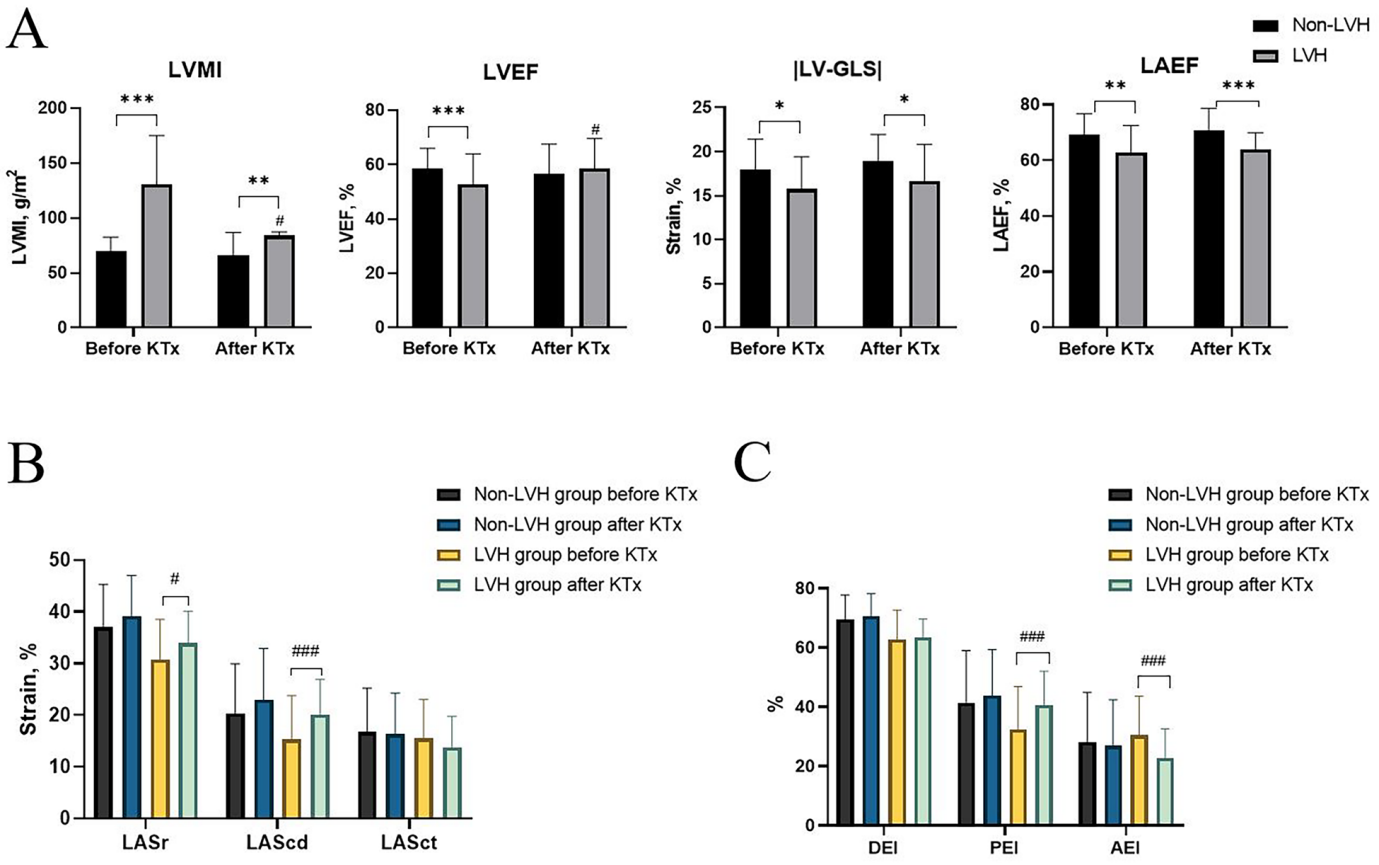
Echocardiographic characteristics before and after KTx among the non-LVH and LVH groups. **(A)** LVMI, LVEF, LV-GLS, and LAEF. **(B)** LASr, LAScd, and LASct. **(C)** DEI, PEI, and AEI. *P < 0.05 (before vs. after KTx), **P < 0.01 (before vs. after KTx), ***P < 0.001 (before vs. after KTx), and #*P* < 0.05 (LVH group vs. non-LVH group after KTx). KTx, kidney transplantation; LVH, left ventricular hypertrophy; LVMI, left ventricular mass index; LV, left ventricle; EF, ejection fraction; LV-GLS, global longitudinal peak systolic left ventricular strain; LA, left atrium; LASr, LAScd, and LASct, left atrial reservoir, conduit, and contraction strain; LAVI, left atrial volume index; DEI, diastolic emptying index; PEI, passive emptying index; AEI, active emptying index.

**Figure 2:**
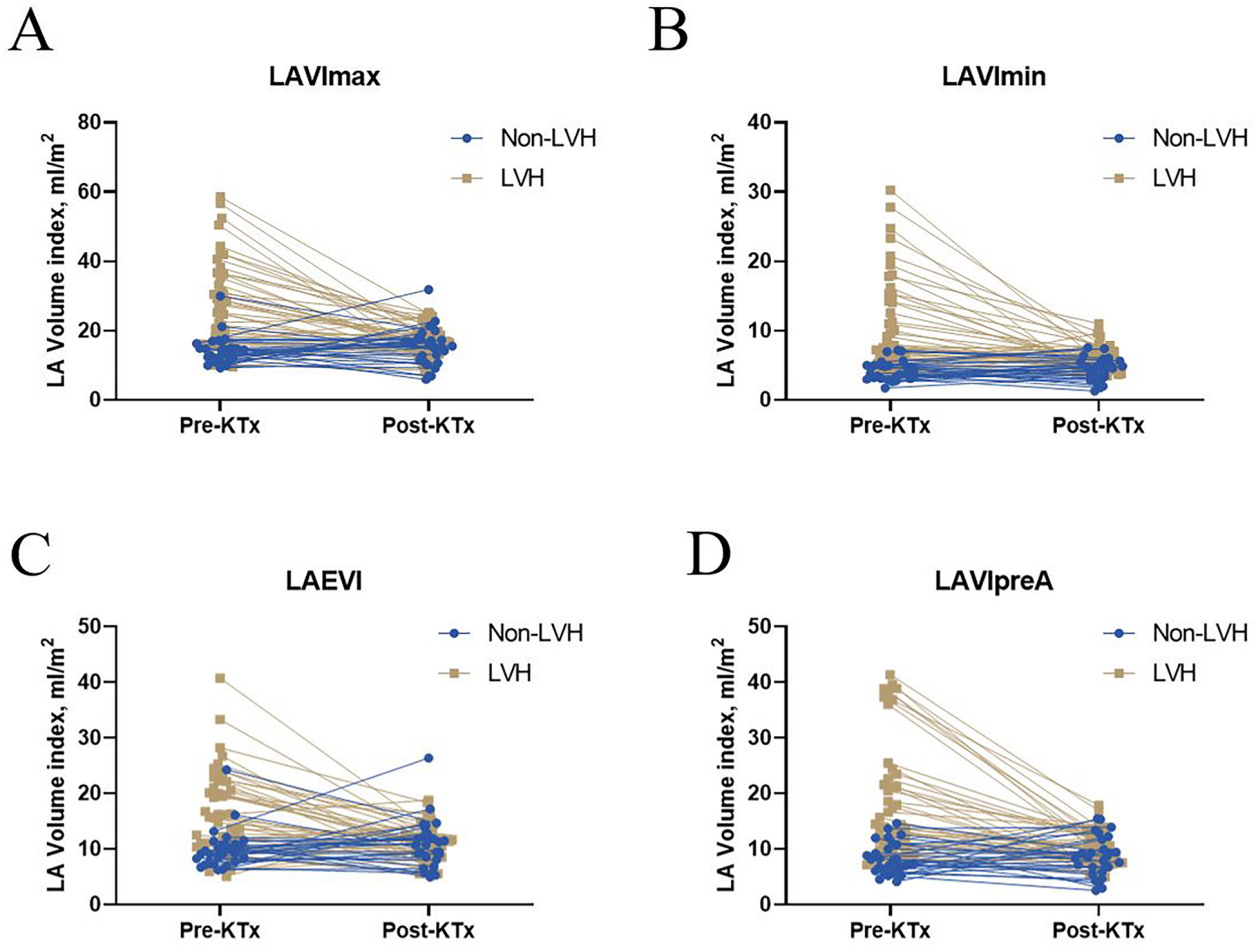
Left atrial volume before and after KTx among the non-LVH and LVH groups. Significant differences in **(A)** LAVImax, **(B)** LAVImin, **(C)** LAEVI, and **(D)** LAVIpreA were found between the before and after KTx values in the LVH group (*P* < 0.001). No significant difference in LAVImax, LAVImin, LAVIpreA, and LAEVI were found between the before and after KTx values in the non-LVH group (*P* > 0.05). KTx, kidney transplantation; LAVImax, maximum left atrial volume index; LAVImin, minimum left atrial volume index; LAEVI, left atrial emptying volume index; LVH, left ventricular hypertrophy; LAVIpreA, volume index before atrial contraction.

LVEF and LVMI were better after transplantation in the LVH group (Figure 1A). LASr and LAScd significantly increased in the LVH group (Figure 1B). Furthermore, after surgery, the LVH group's PEI increased, and the AEI decreased (Figure 1C). After kidney transplantation, the non-LVH group showed no characteristics suggestive of alterations in LA volume, while the LVH group showed a decline in the LAEVI, LAVImax, LAVImin, and LAVIpreA (Figures 2A–D, *p* < 0.001).

### 3.3 Changes in echocardiographic and clinical characteristics before and after KTx according to LVMI categories

After KTx, compared with the recipients in the non-LVH group, favorable changes in SBP, Hb (−11.71 ± 16.27 g/L vs. 25.79 ± 25.13 g/L, *p* = 0.011), E/A, LVMI (−3.63 ± 18.39 g/m^2^ vs. −47.18 ± 38.66 g/m^2^, *p* < 0.001), and LVEF were present in the recipients with LVH, while no statistically significant difference in LV-GLS, LASr, and LAEF were observed (Table 3). The LVH group had a greater decrease in the LAEVI, LAVImax, LAVImin, and LAVIpreA than did the non-LVH group (*p* < 0.001).

**Table 3:** Changes in echocardiographic and clinical characteristics before and after KTx according to left ventricular mass index categories before KTx.

Variables	Non-LVH group (*n* = 28)	LVH group (*n* = 43)	*p-*Value
Clinical characteristics			
△Office SBP (mmHg)	−3.61 ± 13.55	−17.74 ± 20.40	**0**.**002**
△Office DBP (mmHg)	−5.89 ± 14.47	−13.23 ± 16.53	0.059
△HR (bpm)	−0.22 ± 17.65	0.31 ± 19.39	0.918
△Hb (g/L)	11.71 ± 16.27	25.79 ± 25.13	**0**.**011**
Lack of Hb increase	16 (57.1)	13 (30.2）	**0**.**024**
Echocardiographic parameters			
△E/A	−0.18 ± 0.49	0.08 ± 0.53	**0**.**048**
△E/E'	−0.20 ± 1.93	−1.48 ± 3.09	0.055
△LVMI (g/m^2^)	−3.63 ± 18.39	−47.18 ± 38.66	**<0**.**001**
△LV-EDVI (mL/m^2^)	−5.05 ± 38.08	−15.52 ± 36.58	0.250
△LV-ESVI (mL/m^2^)	−1.50 ± 16.73	−12.08 ± 24.81	0.052
△LVEF (%)	−1.67 ± 11.05	5.83 ± 9.22	**0**.**003**
△GLS (%)	−0.89 ± 3.56	−0.92 ± 4.42	0.978
△LASr (%)	2.11 ± 7.47	3.16 ± 9.17	0.612
△LAScd (%)	2.64 ± 9.45	5.00 ± 7.83	0.257
△LASct (%)	−0.43 ± 8.69	−1.74 ± 7.85	0.510
△LAEF (%)	1.64 ± 7.86	1.15 ± 10.73	0.835
△LAEVI (mL/m^2^)	0.89 ± 4.92	−5.89 ± 7.36	**<0**.**001**
△LAVImax (mL/m^2^)	0.84 ± 6.18	−10.58 ± 12.55	**<0**.**001**
△LAVImin (mL/m^2^)	0.09 ± 1.58	−4.56 ± 6.83	**<0**.**001**
△LAVIpreA (mL/m^2^)	0.28 ± 4.41	−8.80 ± 9.91	**<0**.**001**
△DEI (%)	1.06 ± 7.25	0.74 ± 11.36	0.895
△PEI (%)	2.30 ± 17.35	8.38 ± 14.81	0.119
△AEI (%)	−1.24 ± 17.90	−7.64 ± 13.70	0.093

KTx, kidney transplantation; BMI, body mass index; SBP, systolic blood pressure; DBP, diastolic blood pressure; Hb, hemoglobin; LV, left ventricle; LA, left atrium; LVMI, LV mass index; LVH, left ventricular hypertrophy; EDVI, end-diastolic volume index; ESVI, end-systolic volume index; EF, ejection fraction; E and A, mitral peak E- and A-wave velocity; E′, pulsed-wave TDI-derived mitral annular early diastolic velocity; LAVI, left atrial volume index; LASr, LAScd, and LASct, left atrial reservoir, conduit, and contraction strain; LV-GLS, global longitudinal peak systolic left ventricular strain; DEI, diastolic emptying index; PEI, Passive emptying index; AEI, Active emptying index. Bold values indicate that *p*-values < 0.05.

### 3.4 Analysis of HB increase

In this study, 29 (40.8%) recipients did not have an adequate increase in Hb levels, including 16 (57.1%) in the non-LVH group and 13 (30.2%) in the LVH group. The causes of ESKD were unrelated to a lack of Hb increase (Supplementary Table S2). None of the patients treated with the double immunosuppressants experienced inadequate postoperative Hb increase. Tables 4, 5 show the result of univariate and multivariate logistics regression analyses of the factors associated with a lack of Hb increase. Among the candidate variables that were assessed in univariate analysis, factors that were predictive of the absence of a Hb increase in the cohort were included in the multivariable logistic model.

**Table 4:** The logistics regression model analysis of the association between the pre-KTx clinical and echocardiographic parameters and a lack of a Hb increase.

Univariable	OR	95% CI	*β*	*p*-Value
Clinical characteristics				
Duration of CKD (months)	1.002	0.986–1.019	0.002	0.806
Duration of dialysis (months)	1.038	0.998–1.079	0.037	0.062
Ratio of dialysis duration to CKD duration	1.398	0.366–5.345	0.335	0.625
Office SBP (mmHg)	0.972	0.945–1.001	−0.028	0.057
Office DBP (mmHg)	0.961	0.927–0.996	−0.040	**0**.**031**
HR (bpm)	1.013	0.980–1.047	0.013	0.445
LVMI (g/m^2^)	0.981	0.967–0.995	−0.019	**0**.**007**
Echocardiographic parameters				
E/A	1.199	0.415–3.459	0.181	0.738
E/E'	0.717	0.559–0.919	−0.333	**0**.**009**
LV-EDVI (mL/m^2^)	0.976	0.954–0.998	−0.024	**0**.**036**
LV-ESVI (mL/m^2^)	0.964	0.929–1.001	−0.037	0.054
LVEF (%)	1.075	1.013–1.141	0.072	**0**.**017**
GLS (%)	0.858	0.737–0.998	0.154	**0**.**047**
LASr (%)	1.068	1.002–1.138	0.066	**0**.**042**
LAScd (%)	1.010	0.958–1.064	0.010	0.722
LASct (%)	1.064	0.996–1.136	0.062	0.067
LAEF (%)	1.045	0.987–1.107	0.044	0.130
LAEVI (mL/m^2^)	0.928	0.857–1.004	−0.075	0.064
LAVImax (mL/m^2^)	0.954	0.910–0.999	−0.047	**0**.**047**
LAVImin (mL/m^2^)	0.909	0.823–1.004	−0.095	0.059
LAVIpreA (mL/m^2^)	0.957	0.906–1.012	−0.044	0.124
DEI (%)	1.042	0.988–1.100	0.041	0.131
PEI (%)	0.998	0.969–1.028	−0.002	0.898
AEI (%)	1.020	0.987–1.054	0.020	0.246
Multivariable				
LVMI (g/m^2^)	0.981	0.967–0.995	−0.019	**0**.**007**

eGFR, infection, and rejection were adjusted for in the multivariable analysis. KTx, kidney transplantation; SBP, systolic blood pressure; DBP, diastolic blood pressure; Hb, hemoglobin; LV, left ventricle; LA, left atrium; LVMI, LV mass index; LVH, left ventricular hypertrophy; EDVI, end-diastolic volume index; ESVI, end-systolic volume index; EF, ejection fraction; E and A, mitral peak E- and A-wave velocity; E′, pulsed-wave TDI-derived mitral annular early diastolic velocity; LAVI, left atrial volume index; LASr, LAScd, and LASct, left atrial reservoir, conduit, and contraction strain; LV-GLS, global longitudinal peak systolic left ventricular strain; DEI, diastolic emptying index; PEI, passive emptying index; AEI, active emptying index. Bold values indicate that *p*-values < 0.05.

**Table 5:** The logistics regression model analysis of the association between changes in clinical and echocardiographic parameters and a lack of a Hb increase.

Univariable	OR	95% CI	*β*	*p*-Value
Clinical characteristics				
△Office SBP (mmHg)	1.022	0.995–1.050	0.022	0.114
△Office DBP (mmHg)	1.006	0.976–1.036	0.006	0.707
△HR (bpm)	0.994	0.965–1.023	−0.006	0.672
△LVMI (g/m^2^)	1.028	1.009–1.046	0.028	**0**.**004**
Echocardiographic parameters				
△E/A	0.719	0.282–1.833	−0.330	0.490
△E/E'	1.379	1.090–1.746	0.321	**0**.**007**
△LV-EDVI (mL/m^2^)	1.021	1.001–1.041	0.021	**0**.**035**
△LV-ESVI (mL/m^2^)	1.038	1.003–1.074	0.037	**0**.**033**
△LVEF (%)	0.915	0.862–0.971	−0.089	**0**.**003**
△GLS (%)	1.053	0.937–1.185	0.052	0.385
△LASr (%)	0.990	0.935–1.047	−0.010	0.718
△LAScd (%)	1.007	0.952–1.065	0.007	0.798
△LASct (%)	0.984	0.927–1.043	−0.016	0.583
△LAEF (%)	0.996	0.948–1.047	−0.004	0.870
△LAEVI (mL/m^2^)	1.142	1.043–1.251	0.133	**0**.**004**
△LAVImax (mL/m^2^)	1.083	1.023–1.147	0.080	**0**.**006**
△LAVImin (mL/m^2^)	1.139	1.008–1.284	0.130	**0**.**036**
△LAVIpreA (mL/m^2^)	1.075	1.007–1.148	0.072	**0**.**031**
△DEI (%)	0.994	0.947–1.043	−0.006	0.804
△PEI (%)	1.001	0.972–1.031	0.001	0.940
△AEI (%)	0.996	0.967–1.027	−0.004	0.815
Multivariable				
△LVEF (%)	0.912	0.855–0.973	−0.092	**0**.**005**
△LAEVI (mL/m^2^)	1.153	1.041–1.276	0.142	**0**.**006**

eGFR, infection and rejection were adjusted in multivariable analysis; KTx, kidney transplantation; SBP, systolic blood pressure; DBP, diastolic blood pressure; Hb, hemoglobin; LV, left ventricle; LA, left atrium; LVMI, LV mass index; LVH, left ventricular hypertrophy; EDVI, end-diastolic volume index; ESVI, end-systolic volume index; EF, ejection fraction; E and A, mitral peak E- and A-wave velocity; E′, pulsed-wave TDI-derived mitral annular early diastolic velocity; LAVI, left atrial volume index; LASr, LAScd, and LASct, left atrial reservoir, conduit, and contraction strain; LV-GLS, global longitudinal peak systolic left ventricular strain; DEI, diastolic emptying index; PEI, Passive emptying index; AEI, active emptying index. Bold values indicate that *p*-values < 0.05.

The results of univariate logistics regression analysis indicate that DBP, LVMI, E/E’, LV-EDVI, and LAVImax before KTx significantly negatively contributed to a lack of Hb increase. LVEF, LV-GLS, and LASr positively contributed to a lack of Hb increase. With an ROC cutoff of 0.68, the multivariate logistic regression analysis revealed that the LVMI prior to KTx was significantly negatively associated with a lack of Hb increase (*p* = 0.007). In addition, as shown in Table 5, there was a significant positive correlation between the absence of an increase in Hb and changes in LVMI, E/E’, LAEVI, LAVImax, LAVImin, and LAVIpreA. Changes in the LVEF had a significant negative correlation with the absence of an increase in Hb. According to the multivariate logistic regression analysis, which had an ROC cutoff of 0.81, changes in the LVEF and LAEVI contributed negatively and positively to the absence of Hb increase, respectively.

## 4 Discussion

In this study, we found that pediatric recipients with LVH had lower Hb levels before KTx and better Hb levels in the early stage after the KTx therapy. Anemia was more prevalent in the LVH group before KTx than in the non-LVH group. Furthermore, after KTx, there was no discernible difference in anemia between the two groups. Prior to KTx, the non-LVH group had better LV and LA function (LVEF, LV-EDVI, LV-ESVI, LAEVI, LAVImax, LAVImin, and LAVIpreA) and lower LVMI than the LVH group. Before and after KTx, the DEI was lower in the LVH group than in the non-LVH group. After KTx, various echocardiographic parameters were similar between the LVH and non-LVH groups, although the non-LVH group still had better DBP, LV-GLS, LASr, LAVImin, LAEF, and DEI. In comparison to those in the non-LVH group, recipients with LVH demonstrated better changes in Hb, LVMI, SBP, E/A, LVEF, and LA volume parameters after KTx. Importantly, the absence of an Hb increase in the early postoperative period in children was related to pre-KTx LVMI and changes in LVEF and LAEVI before KTx.

It was found that LVH was more common in patients with ESKD and those with anemia (22). Previous research indicated that there was an association between anemia and LVH (23). Xiao et al. observed that the LVMI and blood pressure decreased while the LVEF improved in pediatric recipients after KTx (24). Another study found that post-KTx LV-GLS levels were similar to those during childhood CKD assessment (25). A previous study reported that cardiac magnetic resonance imaging revealed improvements in the LV-ESVI and LV-EDVI post-KTx in adult recipients (26). The findings of our study are consistent with those of the previous studies described.

Hypertension can cause structural and functional heart changes, including interventricular septal thickening, reduced LVEF, and increased LA volume. Our study found a negative correlation between LVMI pre-KTx and changes in LVEF and a positive correlation in changes in the LAEVI with a lack of an increase in postoperative Hb levels. The specific mechanisms linking these measures to blood pressure are still unclear. We plan to explore the relationship between cardiac work and postoperative Hb increases in future research, as this may better illustrate the role of blood pressure in these changes.

In this study, we first showed that the LVH group had a lower DEI than the non-LVH group both before and after KTx. We found that the PEI increased and AEI decreased after KTx in the LVH group. As these are new measures of LA diastolic function (27, 28), the relevant research is limited. However, this study is the first to discuss the DEI, AEI, and PEI of patients before and after KTx. Therefore, multicenter research in a larger cohort is required to validate our findings.

The non-LVH group appeared to be more likely to have a lack of Hb increase than the LVH group during the early postoperative period following transplantation. This observation may be due to the higher pre-KTx Hb levels in the non-LVH group, which suggests that there was less potential for post-KTx Hb levels to increase. In our study, we found that Hb levels were lower in the LVH group than in the non-LVH group before KTx but were similar between the two groups after KTx. Moreover, changes in Hb levels were higher in the LVH group than in the non-LVH group (11.71 ± 16.27 g/L vs. 25.79 ± 25.13 g/L, *p* = 0.011). Therefore, the above conjecture was supported by our results.

Goldman et al. found that the absence of Hb increase at 3 months might have an association with a higher risk of the primary outcome of mortality censored graft failure (7). However, cardiovascular parameters, such as blood pressure, LVMI, and LVEF, were not mentioned in this study, and pediatric recipients were not included. Thus, the population examined in Goldman et al.'s study differed from that in our study. Long-term follow-up is still needed to explore the relationships between Hb increase and left heart functions in pediatric transplant recipients.

In summary, using echocardiographic features, the findings of our study imply that children with ESKD and LVH have a lower level of Hb before KTx and a higher level of Hb increase in the early stage after KTx. In the children, the absence of a Hb increase during the early postoperative period following transplantation was negatively correlated with LVMI before KTx and changes in the LVEF and was positively correlated with changes in the LAEVI. The findings of this study suggest that children without myocardial hypertrophy before KTx, particularly those with anemia, may require more erythropoiesis-stimulating agents or iron supplements to ensure a rapid increase in Hb levels postoperatively. This study has some limitations that merit consideration. First, this was a single-center study with a modest sample size. Therefore, multicenter studies with larger sample cohorts are needed to validate our findings. Furthermore, more blood indices, such as mean corpuscular volume, mean corpuscular hemoglobin concentration, mean corpuscular hemoglobin, and iron profile, which are specific indicators of anemia, should be included in further study.

## Data Availability

The original contributions presented in the study are included in the article/Supplementary Material, further inquiries can be directed to the corresponding authors.
