# Supplementary material for: Left heart function and strain for predicting change in hemoglobin levels in pediatric kidney transplantation recipients
Source: Front Pediatr. 2025 Mar 21;13:1452928. doi: 10.3389/fped.2025.1452928 (PMC11968651; doi:10.3389/fped.2025.1452928)
Supplement: Supplementary file 1 [file Table1.docx]

**Supplemental Table 1.** Characteristics of cause of end-stage kidney disease, infections, acute rejection and ferritin after KTx, and medicine use

|  | Non-LVH group (n = 28) | LVH group (n = 43) | *p* value |
| --- | --- | --- | --- |
| **Cause of end-stage kidney disease, n(%)** |  |  |  |
| CAKUT | 12 (42.9%) | 6 (14.0%) | 0.006 |
| Genetic disease | 5 (17.9%) | 9 (20.9%) | 0.750 |
| Glomerular diseases | 6 (21.4%) | 16 (37.2%) | 0.160 |
| Secondary to systemic diseases | 1 (3.57%) | 7 (16.3%) | 0.135 |
| Others | 4 (14.3%) | 5 (11.6%) | 0.732 |
| **Infections after KTx, n** | 6 (21.4%) | 7 (16.3%) | 0.770 |
| CMV viremia | 0 | 3 | 0.153 |
| B19 Parvovirus | 0 | 1 | 0.416 |
| BK Virus | 2 | 1 | 0.324 |
| Respiratory infections | 1 | 2 | 0.825 |
| Urinary tract infection | 2 | 1 | 0.324 |
| Gastrointestinal Infection | 1 | 0 | 0.212 |
| **Acute rejection after KTx, n(%)** | 2 (7.14) | 3 (6.98) | 0.979 |
| **Ferritin after KTx, μg/L** | 130.87±159.72 | 179.95±269.38 | 0.390 |
| **Medicine use** |  |  |  |
| Iron supplements, n(%) | 22 (78.6) | 28 (65.1) | 0.225 |
| ESA, n(%) | 16 (57.1) | 21 (48.8) | 0.494 |
| Dose of ESA，IU/kg/week | 71.47±70.18 | 64.24±76.07 | 0.688 |
| Antihypertensive medication | 17 (60.7) | 38 (88.4) | 0.006 |

KTx, kidney transplantation; CMV, Cytomegalovirus; CAKUT, Congenital anomalies of the kidney and urinary tract; ESA, erythropoiesis-stimulating agents.

**Supplemental Table 2.** The univariable logistics regression model analysis in characteristics of cause of end-stage kidney disease, infections, acute rejection, ferritin, and medicine use after KTx with lack of Hb increase

|  | OR | 95% CI | *p* value |
| --- | --- | --- | --- |
| **Cause of end-stage kidney disease** | |  |  |
| CAKUT | 0.897 | 0.300-2.677 | 0.845 |
| Genetic disease | 1.109 | 0.340-3.620 | 0.864 |
| Glomerular diseases | 0.573 | 0.199-1.652 | 0.302 |
| Secondary to systemic diseases | 1.520 | 0.348-6.644 | 0.578 |
| Others | 1.979 | 0.483-8.111 | 0.343 |
| **Infections after KTx** |  |  |  |
| CMV viremia | 0.714 | 0.062-8.266 | 0.788 |
| B19 Parvovirus | - | - | - |
| BK Virus | 0.714 | 0.062-8.266 | 0.788 |
| Respiratory infections | 0.714 | 0.062-8.265 | 0.788 |
| Urinary tract infection | 3.037 | 0.262-35.163 | 0.374 |
| Gastrointestinal Infection | - | - | - |
| **Acute rejection after KTx** | 0.963 | 0.151-6.157 | 0.968 |
| **Ferritin after KTx** | 0.999 | 0.998-1.002 | 0.789 |
| **Medicine use** |  |  |  |
| Iron supplements | 1.177 | 0.414-3.344 | 0.760 |
| ESA | 0.771 | 0.299-1.991 | 0.591 |
| Dose of ESA | 0.671 | 0.259-1.738 | 0.411 |
| Antihypertensive medication | 0.618 | 0.201-1.895 | 0.399 |

KTx, kidney transplantation; CMV, Cytomegalovirus; CAKUT, Congenital anomalies of the kidney and urinary tract; ESA, erythropoiesis-stimulating agents.
